# Structuring and advancing solution-oriented research for sustainability: *This article belongs to* Ambio*’s50th Anniversary Collection. Theme: Solutions-oriented research*

**DOI:** 10.1007/s13280-021-01537-7

**Published:** 2021-03-14

**Authors:** Daniel J. Lang, Arnim Wiek

**Affiliations:** 1 Faculty of Sustainability, Leuphana University of Lüneburg, Universitätsallee 1, 21339 Lüneburg, Germany; 2 Center for Global Sustainability and Cultural Transformation, Leuphana University of Lüneburg, Lüneburg, Germany; 3 Center for Global Sustainability and Cultural Transformation, Arizona State University, Tempe, AZ USA; 4 School of Sustainability, Arizona State University, Tempe, AZ USA

**Keywords:** Connection of research efforts, Cross-case comparisson, Enabling actions, Leverage points, Opportunities for advancement, Typology

## Abstract

The sustainability challenges the world faces today call for concerted and immediate action. Complementing problem-oriented, descriptive-analytical research with solution-oriented research could strengthen sustainability science’s contribution to address these challenges. We introduce different types of solution-oriented sustainability research to structure the discourse, outline opportunities to advance this research trajectory, and close with recommendations on how to support particularly students and early career researchers in getting involved with solution-oriented sustainability research.

## Introduction

Sustainability challenges such as climate change, loss of biodiversity, economic injustice, or spreading diseases threaten viability and integrity of nature and society around the world. There is a need for concerted and immediate action to address these challenges. Research can and needs to contribute to designing, testing and evaluating these actions. In the past, researchers have provided profound insights into the complex structures and dynamics of social-ecological systems, as well as the concomitant sustainability challenges. While most researchers continue to focus on this problem-oriented, descriptive-analytical research trajectory, some research efforts have shifted to studying and advancing solution options (Wiek et al. 2012; Miller et al. 2014). The five *Ambio* articles reprinted in this anniversary collection can be seen as precursors of this solution-oriented research trajectory. Reviewing the current sustainability research landscape, it seems that these two trajectories could be better connected and complement each other. We focus here on the solution-oriented trajectory and first introduce different types of solution-oriented sustainability research to highlight connecting points; second, identify areas for further advancement of solution-oriented research; and third, explore how solution-oriented research can be effectively supported.

## Types of solution-oriented sustainability research

Solution-oriented sustainability research investigates actions and practices—called “solution options” in the following—that are intended to advance sustainable development. Research on solution options can be differentiated temporally (*when* the research is undertaken) as well as regarding the role of the researcher in implementing solution options. Both dimensions shape the way this research is conducted and the results it yields. First, solution options can be studied (1) *before* their implementation (ex-ante) to appraise potential impacts of the solution options (when implemented) and to inform their design (Culotta et al. 2016); (2) *during* their implementation (in situ) to explore their initial effectiveness (while implemented) and inform necessary adaptations (Caniglia et al. 2017); or (3) *after* their implementation (ex-post) to evaluate their overall effectiveness and transferability (Luederitz et al. 2017). Ideally, these three approaches are conducted in an iterative sequence (see below). Second, researchers can (1) collaborate with potential implementers (e.g. policy makers or NGOs) as well as affected actors on developing, implementing, and evaluating solution options through transdisciplinary research (Lang et al. 2012); or, alternatively, (2) take a more distant and unidirectional approach, i.e. informing or consulting with implementers and affected actors (Wiek 2007; Spangengberg 2011).

Combining these two dimensions, six types of solution-oriented sustainability research can be differentiated:

1. *Ex-ante & distant*: In this type, scientists develop new solution options through unidirectional interactions (information and/or consultation) with future implementers and potentially affected actors. Examples range from sustainable chemistry research to design substances that do not harm environment and people (Kümmerer et al. 2020) to research on policies or programs that incentivize greenhouse gas emission reductions (Liu et al. 2019).
2. *Ex-ante & engaging*: Here, scientists collaborate with potential implementers as well as potentially affected actors in planning and designing solutions in bi-directional ways. Examples include transdisciplinary scenario construction to develop solution options in complex systems (Oteros-Rozas et al. 2015), collaborative development of an intervention plan to reach a sustainability vision (Bernstein et al. 2016), or exploration of solution options in decision visualization environments (John et al. 2020).
3. *In situ & distant*: In this type, scientists test solution options within controlled settings. A prominent example are randomized-control trials to test the effectiveness of different policy options for poverty alleviation (Banerjee and Duflo 2011). These experiments generate evidence on how solution options perform under real-world conditions.
4. *In situ & engaging*: Here, scientists and potential implementers actively collaborate on experiments to test solution options. In contrast to (3), solution options as well as experimental designs are created collaboratively. Exemplary settings for such experiments are real-world labs (Schäpke et al. 2018) or urban living labs (Voytenko et al. 2016).
5. *Ex-post & distant*: In this type, solution options are studied after the implementation (ex-post). This offers the potential to identify impacts and generalizable patterns of success or failure. Examples include research on successful schemes for governing the commons (Ostrom 2009), or the compilation of Seeds of a Good Anthropocene (Bennett et al. 2016).
6. *Ex-post & engaging*: Here, implementers and affected actors collaborate in evaluating solution options’ implementation to improve their outcomes through adaptation or to explore their transferability. For example, Forrest et al. (2020) collaboratively evaluated the transferability of transformational water solutions.

Most of the contributions reprinted in this anniversary edition of *Ambio* relate to the “distant” types of solution-oriented research, but some also hint towards more engaged forms of research. Falkenmark (1989), for instance, analyzes water scarcity problems in Africa and highlights intervention points to overcome these problems. From this problem-oriented research, ex-ante policy design and experimentation could emerge. The study closes with a call for “developing indigenous expertise by giving high priority to research, education and training” (Ibid. p. 118), which points to the *in situ & engaging* type. Similarly, Nepstad et al. (1991) identify ecological barriers to forest regrowth in the Amazon and outline strategy components to overcome these barriers, paving the way for *ex-ante and in situ* experimentations. Cassman et al. (2002) outline a research and policy agenda for nitrogen management to contribute “meeting increased food demand and protecting environmental nature” (Ibid. p. 132). As part of their research priorities, they call for *in situ & distant/engaged* experimentation as “quantitative on-farm evaluations of improved technologies and measurements of N losses” (Ibid. p 139). Both Folke and Kautsky (1989) as well as Brix and Schierup (1989) can be seen as *ex-post & distant* evaluations. While Folke and Kautsky (1989) comparatively evaluate two aquaculture system to derive recommendations for an “ecologically integrated technology and sustainable aquaculture” (Ibid. p. 242), Brix and Schierup (1989) evaluate the implementation of aquatic macrophytes in water-pollution control.

## Advancing solution-oriented sustainability research

There are several opportunities to advance solution-oriented sustainability research with regards to both its effectiveness in creating societal impact as well as its ability to broaden the scientific knowledge base. Some opportunities pertain to a specific type, others cut across several types. We introduce three opportunities below that connect to our own research hoping they might also inspire novel contributions in *Ambio*.

### Focusing on deep leverage points and complexity

The first opportunity pertains to research on leverage points, i.e. “places in complex systems where a small shift may lead to fundamental changes in the system as a whole” (Abson et al. 2017, p. 30), to foster sustainability transformation. A focus area is research on *deep* leverage points, such as changing values as levers for fundamental changes in system structures and dynamics (Horcea-Milcu et al. 2019). Advancing our understanding, both generally and in specific contexts, where to intervene effectively to foster fundamental systems change informs targeted *ex-ante as* well as *in situ experimentation*. In return, such experimentation as well as *ex-post evaluation* of solution implementations can increase the understanding of leverage points.

### Cross-case comparisons to transfer and scale solution options

The second opportunity relates to coordinated research across *in situ experiments* as well as *ex-post evaluations* of implemented solution options. Such studies can be based, for example, on a stratification strategy for several experiments (Engler et al. 2019), a unified evaluation scheme (Luederitz et al. 2017), or a real-world laboratory for continuous experimentation (Bergmann et al. 2021). Adequately designed, these cross-case comparisons help linking context-specific to generic insights (Wiek et al. 2012; Withycombe Keeler et al. 2016). This, in return, creates the basis for studies on amplifying the impact of solution options (Lam et al. 2020) through transfer and scaling ex-ante or in situ.

### Connecting research efforts to advance solutions

The third opportunity pertains to *connecting* different research efforts, including:

(i) Connecting different types of solution-oriented sustainability research, for instance, through integrative research methodologies such as the TRANSFORM framework (Wiek & Lang 2016). As indicated above, research of the different types can inform each other to develop and implement comprehensive and effective solution options.
(ii) Connecting problem-oriented and solution-oriented sustainability research approaches, for example, by linking modeling/simulation of complex social-ecological systems, which provides increasingly detailed insights into system structures and dynamics on a generic level, to real-world experimentation *(ex-ante and in situ),* which can translate these insights into context-dependent settings and provide evidence how to improve and validate the models (Lang et al. 2017).
(iii) Connecting different fields of solution-oriented research, from intervention research in public health to behavioral economics and positive psychology to generate evidence for interventions that foster sustainable behavior (e.g. Robinson & Sirard 2005).
(iv) Connecting solution-oriented sustainability research to local or indigenous knowledge generation to broaden the spectrum of solution options. This connection is further explored in Andersson & Tengö (2021—in this volume).

We use the term “connecting” here to recognize the need for different epistemologies and approaches to investigate complex systems and act in them. Such “integrative pluralism” (Mitchell 2009) calls for meta-level research to further develop theories and methodologies that help to utilize the diversity of research approaches that foster sustainability.

## Enabling solution-oriented sustainability research

To seize the opportunities outlined above and foster solution-oriented sustainability research requires significant institutional changes (Van der Leeuw et al. 2012; Miller et al. 2014). For instance, despite strong personal interest, students and early career researchers are often reluctant to engage in this research trajectory because of institutional barriers. The following four actions could help overcome such barriers:

- Capacity building in solution-oriented research on all academic levels, from undergraduate, graduate, post-graduate to continuing education for advanced researchers to enable broad engagement with solution-oriented research and to realize the aforementioned connections in mutually appreciative ways.
- Changes in the academic reward system to incentivize solution-oriented research and the aforementioned connections, which might require additional efforts and not immediate yield conventional outputs (publications, funds).
- Outline career paths for early career researchers interested in solution-oriented sustainability research including supporting tenure and promotion policies that acknowledge achievements beyond conventional outcomes.
- Publication outlets, such as a specific section in *Ambio*, that create spaces for solution-oriented research and allow for a scientific discourse to advance theoretical underpinning, methodological approaches, and empirical insights.

These recommendations are not entirely new and some progress has been made. However, the limited time to take action on critical Sustainable Development Goals or the need for rapid responses to global crises call for advancing our research practice to keep up with the challenges of societal transformation.

## References

[CR1] Abson DJ, Fischer J, Leventon J, Newig J, Schomerus T (2017). Leverage points for sustainability transformation. Ambio.

[CR2] Banerjee, A.V., and E. Duflo. 2011. *Poor economics: A radical rethinking of the way to fight global poverty*. Public Affairs.

[CR3] Bennett EM, Solan M, Biggs R, McPhearson T, Norström AV (2016). Bright spots: seeds of a good Anthropocene. Frontiers in Ecology and the Environment.

[CR4] Bergmann, M., N. Schäpke, O. Marg, F. Stelzer, D.J. Lang, M. Bossert, M. Gantert, E. Häußler, et al. 2021. Transdisciplinary sustainability research in real-world labs: success factors and methods for change. *Sustainability Science*. 10.1007/s11625-020-00886-8.

[CR5] Bernstein MJ, Wiek A, Brundiers K, Pearson K, Minowitz A, Kay B, Golub B (2016). Mitigating urban sprawl effects – a collaborative tree and shade intervention in Phoenix, Arizona, USA. Local Environment.

[CR6] Brix H, Schierup HH (1989). The use of aquatic macrophytes in water-pollution control. Ambio.

[CR7] Caniglia G, Schäpke N, Lang DJ, Abson DJ, Luederitz C (2017). Experiments and evidence in sustainability science – A typology. Journal of Cleaner Production.

[CR8] Cassman KG, Dobermann A, Walters DT (2002). Agroecosystems, nitrogen-use efficiency, and nitrogen management. Ambio.

[CR9] Culotta D, Wiek A, Forrest N (2016). Selecting and coordinating local and regional climate change interventions. Environment and Planning C: Government and Policy.

[CR10] Horcea-Milcu AI, Abson DJ, Apetrei CI, Duse IA, Freeth R (2019). Values in transformational sustainability science: four perspectives for change. Sustainability Science.

[CR11] Engler JO, Zimmermann H, Lang DJ, Feller RL, von Wehrden H (2019). Towards more effective and transferable transition experiments: learning through stratification. Sustainability Science.

[CR12] Falkenmark M (1989). The massive water scarcity now threatening Africa: why isn’t it being addressed?. Ambio.

[CR13] Folke C, Kautsky N (1989). The role of ecosystems for a sustainable development of aquaculture. Ambio.

[CR14] Forrest N, Stein Z, Wiek A (2020). Transferability and scalability of sustainable urban water solutions—a case study from the Colorado River Basin. Resources, Conservation and Recycling.

[CR15] John, B., D.J. Lang, H. von Wehrden, R. John, and A. Wiek. 2020. Advancing decision-visualization environments—Empirically informed design recommendations. *Futures* 123: 102614.

[CR16] Kümmerer K, Clark JH, Zuin VG (2020). Rethinking chemistry for a circular economy. Science.

[CR50] Lam, D.P.M., B. Martín-López, A. Wiek, E.M. Bennett, N. Frantzeskaki, A.I. Horcea-Milcu, and D.J. Lang. 2020. Scaling the impact of sustainability initiatives – a typology of amplification processes. *Urban Transformations* 2: 3.

[CR17] Lang DJ, Wiek A, Bergmann M, Stauffacher M, Martens P (2012). Transdisciplinary research in sustainability science: practice, principles, and challenges. Sustainability Science.

[CR18] Lang DJ, Wiek A, von Wehrden H (2017). Bridging divides in sustainability science. Sustainability Science.

[CR19] Liu X, Shen B, Price L, Hasanbeigi A, Lu H (2019). A review of international practices for energy efficiency and carbon emissions reduction and lessons learned for China. Wiley Interdisciplinary Reviews—Energy and Environment.

[CR20] Luederitz C, Schäpke N, Wiek A, Lang DJ, Bergmann M (2017). Learning through evaluation—a tentative evaluative scheme for sustainability transition experiments. Journal of Cleaner Production.

[CR21] Miller TR, Wiek A, Sarewitz D, Robinson J, Olsson L (2014). The future of sustainability science—a solutions-oriented research agenda. Sustainability Science.

[CR22] Mitchell SD (2009). Unsimple truths: Science, complexity, and policy.

[CR23] Nepstad DC, Uhl C, Serrao EA (1991). Recuperation of a degraded Amazonian landscape—forest recovery and agricultural restoration. Ambio.

[CR24] Ostrom E (2009). A general framework for analyzing sustainability of social-ecological systems. Science.

[CR25] Oteros-Rozas, E., B. Martín-López, T.M. Daw, E.L. Bohensky, J.R.A. Butler, R. Hill, J. Martin-Ortega, A. Quinlan, et al. 2015. Participatory scenario planning in place-based social-ecological research: Insights and experiences from 23 case studies. *Ecology and Society* 20: 32.

[CR26] Robinson TN, Sirard JR (2005). Preventing childhood obesity: a solution-oriented research paradigm. American journal of preventive medicine.

[CR27] Schäpke N, Stelzer F, Caniglia G, Bergmann M, Wanner M (2018). Jointly experimenting for transformation? Shaping real-world laboratories by comparing them. GAIA—Ecological Perspectives for Science and Society.

[CR28] Spangenberg JH (2011). Sustainability science—A review, an analysis and some empirical lessons. Environmental Conservation.

[CR29] Van der Leeuw S, Wiek A, Harlow J, Buizer J (2012). How much time do we have? Urgency and rhetoric in sustainability science. Sustainability Science.

[CR30] Voytenko Y, McCormick K, Evans J, Schliwa G (2016). Urban living labs for sustainability and low carbon cities in Europe: Towards a research agenda. Journal of Cleaner Production.

[CR31] Wiek A, Lang DJ, Heinrichs H, Martens P, Michelsen G, Wiek A (2016). Transformational Sustainability Research Methodology. Sustainability Science—An Introduction.

[CR32] Wiek A, Ness B, Schweizer-Ries P, Brand FS, Farioli F (2012). From complex systems analysis to transformational change: A comparative appraisal of sustainability science projects. Sustainability Science.

[CR33] Wiek A (2007). Challenges of transdisciplinary research as interactive knowledge generation—experiences from transdisciplinary case study research. GAIA—Ecological Perspectives for Science and Society.

[CR34] Withycombe Keeler L, Wiek A, Lang DJ, Yokohari M, van Breda J, Olsson L, Ness B, Morato J (2016). Utilizing international networks for accelerating research and learning in transformational sustainability science. Sustainability Science.

